# Supplementary material for: The Immunomodulatory CEA Cell Adhesion Molecule 6 (CEACAM6/CD66c) Is a Protein Receptor for the Influenza A Virus
Source: Viruses. 2021 Apr 21;13(5):726. doi: 10.3390/v13050726 (PMC8143321; doi:10.3390/v13050726)
Supplement: Supplementary file 1 [file viruses-13-00726-s001.zip › viruses-1188729-supplementary.pdf]

viruses

Supplementary Materials:

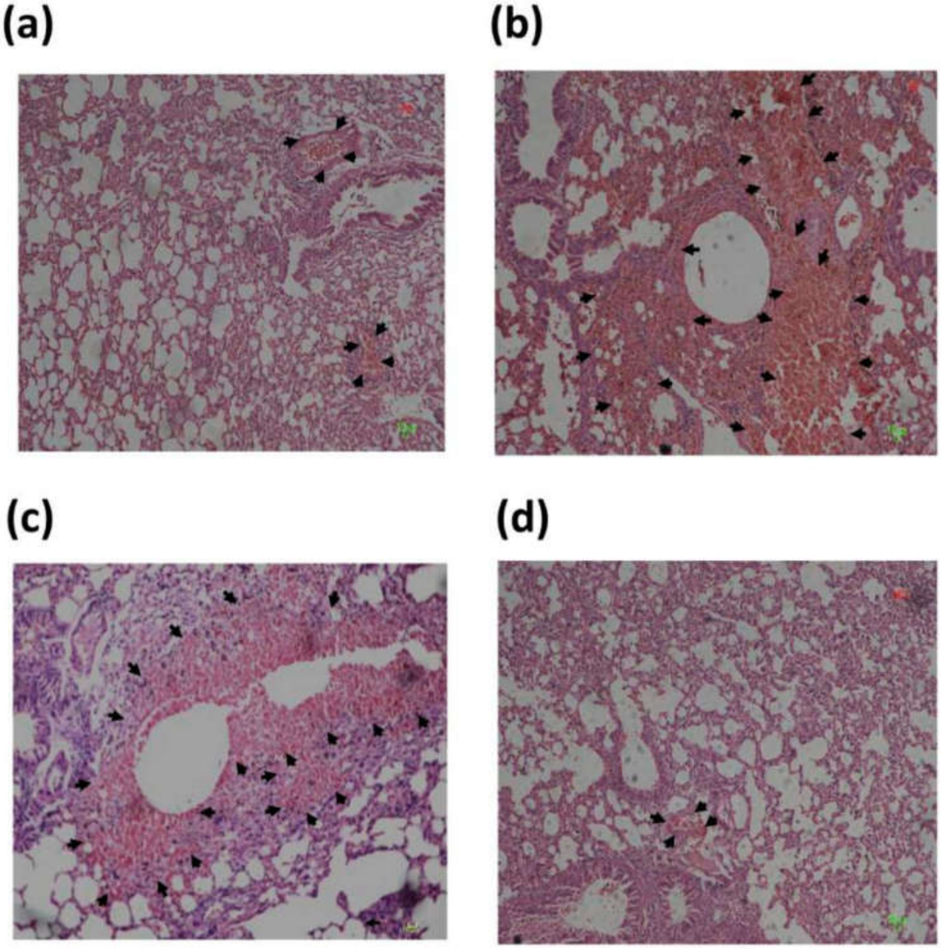

Figure S1: PR8 virus pre-incubated with purified recombinant CD66c causes lower inflammatory response in mice lung. Figure shows histopathological analysis of lung tissues of mice infected with A/PR8/34 influenza virus. Images shown in the figure are lung tissues of sacrificed animals, preserved in formalin, embedded in paraffin and sectioned into serial 4-µm sections. The figure shows images of infected tissues stained with hematoxylin & eosin dye (H&E) captured at 20X magnification. The representative images of mice from different experimental animal groups are — (a) the lung tissues from mice infected with A/PR8/34 virus pre-incubated with CD66c that showed a mild alveolitis and slight hemorrhage (indicated with arrow). (b) H&E stained lung tissues from mice infected with A/PR8/34 virus, showing significant alveolitis and extensive intra-alveolar hemorrhage (marked by arrow). (c) H&E stained lung tissues from mice infected with A/PR8/34 virus pre-incubated with BSA (as a mock protein control), showing alveolitis and intra-alveolar hemorrhage (marked by arrow). (d) Lung tissues from uninfected mice showing no sign of alveolitis.
